# Barriers to Parental Engagement in NICU Infants with Congenital Anomalies: An Exploratory Pilot Study in Fetal Center Mother–Infant Dyads

**DOI:** 10.1055/a-2920-8381

**Published:** 2026-08-11

**Authors:** Jaclyn Ruggiero, Aditi Chunduru, Wen Li, Thomas Cunningham, Elisa Garcia, Alexandra Patch, Jerrie S. Refuerzo, Allison Davidson

**Affiliations:** 1 Division of Neonatal-Perinatal MedicineDepartment of Pediatrics 12339 University of Texas McGovern Medical SchoolHoustonTexasUnited States; 2 Division of Clinical and Translational SciencesDepartment of Internal Medicine 12339 University of Texas McGovern Medical SchoolHoustonTexasUnited States; 3 Division of Fetal InterventionDepartment of Obstetrics, Gynecology and Reproductive Sciences 12339 University of Texas McGovern Medical SchoolHoustonTexasUnited States

**Keywords:** parental visitation, congenital anomalies, skin-to-skin, maternal mental health, expressed breast milk, fetal center

## Abstract

**Objective** This study aimed to explore potential medical, sociodemographic, and maternal mental health (MMH) factors associated with parental engagement in the neonatal intensive care unit (NICU) among mother–infant dyads, followed through a fetal center (FC).

**Study Design** This exploratory pilot study used mixed retrospective and prospective data collection at a Level IV NICU and affiliated FC between June and August 2023. The primary outcome was cumulative parental bedside presence during the first postnatal month. Secondary outcomes included time to first parental visitation, skin-to-skin (STS) care, expressed breast milk (EBM) provision, and MMH indicators. Maternal and infant clinical and sociodemographic variables were retrospectively abstracted from the electronic medical record, while parental visitation patterns were prospectively tracked using bedside documentation tools.

**Results** Sixteen mother–infant dyads were included. Mean time to the first parental visitation was 7.6 h, and mean time to first STS contact was 8.3 days. Exploratory analysis identified patterns suggesting differences in parental engagement by language, insurance status, and MMH indicators. African American parents visited earlier than Caucasian and Hispanic parents ( *p* = 0.05). Descriptively, English-speaking and privately insured families initiated STS earlier than Spanish-speaking and publicly insured families. Earlier parental visitation was also observed among mothers providing EBM at postnatal day 14 ( *p* = 0.016). Mothers prescribed psychotropic medications experienced longer delays to first visitation ( *p* = 0.046). Given the small sample size and exploratory design, findings should be interpreted cautiously.

**Conclusion** In this exploratory pilot cohort of FC mother–infant dyads, parental engagement patterns appeared to vary across sociodemographic and MMH-related factors. These preliminary findings support the feasibility and importance of larger prospective studies examining structural and psychosocial barriers to early parental participation in high-risk NICU populations.

## Introduction

Congenital anomalies are a leading cause of neonatal morbidity and mortality worldwide, affecting approximately 3 to 6% of all live births and contributing significantly to long-term pediatric disability. These anomalies include structural malformations and functional birth defects that account for an estimated 240,000 neonatal deaths annually worldwide. Resource-constrained families are disproportionately affected, with 94% of severe congenital disorders occurring in low- and middle-income countries. 1 In the United States, birth defects affect 3% of infants and remain a major contributor to infant mortality. Congenital malformations were the leading cause of infant deaths in 2022, responsible for 19.5% of all infant deaths. 2 3 As diagnostic capabilities and prenatal imaging improve, more congenital conditions are identified in utero, enabling early intervention planning and coordinated perinatal management. 4 5 The burden of these anomalies extends beyond medical complexity, deeply impacting family systems through prolonged hospitalization, uncertainty, and emotional stress. 6 Within this context, admission to the neonatal intensive care unit (NICU) presents a further disruption to the biologic expectations within the infant–parent dyad. 7

Early parent–infant bonding is essential for neurodevelopment, emotional regulation, and long-term health outcomes. Newborns are neurologically wired for continuous physical and emotional closeness with a primary caregiver, fostering connection and physiologic stability. 7 8 Parent–infant co-regulation plays a crucial role in shaping the developing brain, modulating autonomic nervous system function, and supporting social–emotional development. 7 9 10 Early disruptions in this dyadic interaction, such as parental separation due to NICU admission, can contribute to toxic stress, dysregulation of the hypothalamic–pituitary–adrenal axis, and long-term developmental consequences. 7 10 11 Infants who experience prolonged separation from their parents may exhibit alterations in cortisol secretion, differences in brain connectivity, and delays in developmental milestones. 8 11 Conversely, interventions that promote early and sustained parental presence, including skin-to-skin (STS) care, have been associated with improved neurodevelopmental and physiological outcomes. 12 13 14 15

Despite the well-documented benefits of parental engagement, NICU hospitalization inherently disrupts the normal biological expectations of both the infant and the caregiver. 7 9 Parents of NICU infants frequently experience high levels of psychological distress, including anxiety, depression, and trauma symptoms. 16 17 18 Maternal depression rates in the NICU range from 20% to 50%, and anxiety levels can be similarly elevated. 16 This distress can impair parental engagement, reducing visitation frequency, limiting STS contact, and dampening breastfeeding success. 18 19 Understanding the barriers to parental presence in the NICU is essential to developing targeted interventions that optimize parent–infant interaction and improve long-term outcomes. 13 14 20 21

Fetal centers (FCs) have emerged as a specialized advancement in perinatal medicine, dedicated to supporting families facing high-risk pregnancies involving congenital anomalies. These multidisciplinary programs integrate maternal–fetal medicine, fetal intervention, neonatology, pediatric surgery, genetics, and social work to provide comprehensive prenatal diagnosis, counseling, and coordinated delivery planning. 5 6 Infants followed through an FC are often admitted to the NICU immediately after delivery because of their underlying medical conditions, making opportunities for early parental bonding especially important. Parents may face unique stressors, including prolonged maternal recovery and emotional trauma from the prenatal diagnosis, that challenge bonding and bedside presence. 16 22

While previous research has examined parental stress, bonding, and engagement in traditional NICU populations, most studies have focused on generalized neonatal critical illness and stress arising after birth. 11 16 20 In contrast, families followed through an FC may experience psychosocial stress beginning prenatally with the diagnosis of a congenital anomaly and continuing through the NICU hospitalization. 5 6 22 Anticipatory grief, uncertainty regarding prognosis, complex delivery planning, maternal–fetal monitoring, and preparation for potential surgical intervention may shape parental engagement before the infant is born. As a result, FC families may experience relational and caregiving hardship that differs from those encountered in more traditional NICU populations following unexpected preterm birth or acute neonatal illness. 7 10 22

Although the benefits of parental presence are well-established, the factors associated with early parental engagement among FC families remain poorly understood, particularly across the continuum from prenatal diagnosis through NICU hospitalization. To our knowledge, no studies have specifically examined how psychosocial, socioeconomic, and maternal mental health (MMH) factors relate to parental engagement within this population. 4 21 23 Therefore, this exploratory pilot study aims to address these gaps by examining potential medical, socio-demographic, and MMH-related factors associated with parental engagement among FC mother-infant dyads admitted to the NICU. Specifically, we explored patterns of parental visitation, STS contact, and expressed breast milk (EBM) provision during the early postnatal period.

## Materials and Methods

### Study Design and Setting

This study was a mixed retrospective and prospective study conducted at the Children’s Memorial Hermann Hospital (CMMH) and the affiliated UTHealth Houston Fetal Center (UTH FC) between June and August 2023. The CMHH is a level IV NICU providing specialized care for critically ill neonates. The UTH FC is a high-volume regional referral center that serves as a national multidisciplinary referral center for comprehensive care of high-risk pregnancies complicated by congenital anomalies.

This study was reviewed and approved by the Institutional Review Board of the University of Texas Health Science Center at Houston (HSC-MS-23-0395). Waiver of consent was granted due to the minimal risk nature of the study and the use of routinely collected clinical and observational data. The study was conducted in accordance with institutional and federal ethical standards for human subjects’ research.

### Study Population

This study included mother–infant dyads in which the infant was prenatally diagnosed with a congenital anomaly, the mother received prenatal care at the UTH FC, and the infant was delivered at CMHH and subsequently admitted to the CMHH NICU. Congenital anomaly was operationally defined as a prenatally diagnosed structural or functional fetal abnormality requiring evaluation and coordinated perinatal management through the FC. No exclusions were made based on organ system or severity of anomaly. Exclusion criteria included outborn infants, cases with missing parental visitation data, and infants who did not survive beyond the immediate postnatal period. A convenience sample of eligible mother–infant dyads admitted during the study period between June and August 2023 was included for analysis.

### Data Collection

This exploratory pilot study used both prospective and retrospective data collection methods. Daily delivery logs and NICU admission records were reviewed within the electronic medical record (EMR) to identify eligible dyads.

Parental visitation patterns and initiation of STS care were prospectively tracked during the infant’s hospitalization using bedside documentation calendars. These calendars were developed for this pilot study to prospectively document parental bedside presence and STS initiation. After identifying eligible infants, a study team member placed a calendar at the bedside shortly after NICU admission and checked in multiple days each week with bedside nursing staff regarding documentation and patient disposition plans. Bedside nursing staff documented parental presence and STS activities during routine clinical care, and calendars were collected at discharge for data abstraction. The calendars were not previously validated and were used as a pragmatic observational tool to support prospective tracking of parental presence during this pilot study.

Additional data were retrospectively abstracted from the EMR, including maternal sociodemographic variables (age, race/ethnicity, insurance type, primary language), EBM provision at postnatal day 14, mode of delivery (cesarean vs. vaginal), and whether postnatal surgical interventions were performed. Given the exploratory nature of this study and the inconsistent availability of standardized postnatal mental health screening data, MMH was assessed using clinically documented indicators, including psychotropic medication prescriptions and referrals to perinatal psychiatry services. Edinburgh Postnatal Depression Scale (EPDS) screening results were included when available. However, EPDS data were incomplete across the cohort, so psychotropic medication prescriptions and perinatal psychiatry referrals served as the primary MMH indicators for exploratory analyses.

### Outcome Measures

The primary outcome of this study was cumulative parental time at bedside following NICU admission, recorded weekly for the first four postnatal weeks. Group comparisons were conducted for race/ethnicity, insurance type, primary language, and MMH status to identify disparities in timing of parental visitation, STS initiation, and EBM provision.

Secondary outcomes included time to first parental visitation (measured in hours after birth), time to first parental STS contact (measured in days), presence or absence of EBM provision at postnatal day 14, and MMH indicators, including available EPDS scores, psychotropic medication prescriptions, and referrals to perinatal psychiatry services.

Time to first STS contact was recorded regardless of the clinical reason for delay. Because this was an observational pilot study, infant eligibility for STS based on medical stability, postoperative status, respiratory support requirements, or congenital anomaly type was neither standardized nor used as an exclusion criterion.

Potential barriers to parental presence were categorized into three domains: infant medical characteristics, maternal sociodemographic factors, and MMH. Infant medical characteristics included mode of delivery, type of congenital anomaly, presence of postnatal surgical interventions, mode and duration of respiratory support, oxygen requirement, use of vasopressors for hemodynamic instability, presence of central lines, and presence of intraventricular hemorrhage (IVH). Maternal sociodemographic factors assessed were gravida, parity, age, race, ethnicity, primary language, insurance type, level of education, zip code, number of other children, and length of hospital stay. MMH was evaluated using EPDS scores collected during prenatal FC visits and postnatal hospitalization, as well as clinically documented psychotropic medication prescriptions and referrals to perinatal psychiatry services. Because EPDS screening data were incomplete, psychotropic medication prescriptions and psychiatry referrals served as the primary MMH indicators in exploratory analyses.

### Statistical Analysis

Descriptive statistics were used to summarize demographic characteristics and outcome measures. Continuous variables, including time to first parental visitation and time to first STS contact, were summarized using means and standard deviations (SDs). Given the small sample size, we established an a priori threshold of at least four observations per group for inferential testing; comparisons in which any group contained fewer than four observations were reported descriptively only. For comparisons meeting this threshold, group differences were assessed using *t* tests or analysis of variance (ANOVA), with 95% confidence intervals (CIs) for mean differences. Each parametric comparison was accompanied by a non-parametric sensitivity analysis (Wilcoxon’s rank-sum or Kruskal–Wallis test). Distributions of continuous outcomes were additionally examined visually using violin plots with embedded boxplots to assess variability and skewness. Given the small sample, *p* -values are interpreted as descriptive flags rather than confirmatory evidence, and all analyses are considered exploratory and hypothesis-generating. Given the pilot and exploratory nature of this study, no multiple testing adjustment was conducted.

## Results

**Fig. 1:**
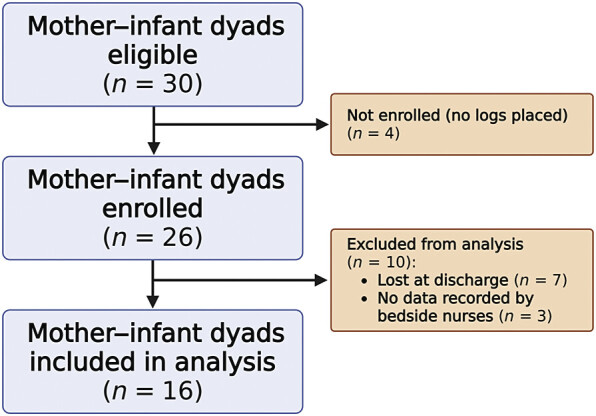
Study flow diagram showing inclusion and exclusion of mother–infant dyads.

A total of 16 mother–infant dyads met inclusion criteria for this study, conducted over a 10-week period from June to August 2023. All infants received prenatal care at the UTH FC and were prenatally diagnosed with one or more congenital anomalies. Of the initial 30 eligible infants, 4 were not enrolled because bedside visitation calendars were not placed at admission, leaving 26 infants successfully enrolled. Of these, 7 bedside calendars were unavailable at discharge, and 3 had incomplete documentation, resulting in 16 dyads included in the final analysis. The study flow, including reasons for exclusion, is shown in Fig. 1 . The primary outcome of cumulative parental bedside presence over the first postnatal month averaged 88.4 h (SD = 61.2).

Infants presented with a range of congenital anomalies, most commonly gastrointestinal anomalies (e.g., gastroschisis, omphalocele, bowel obstruction), followed by thoracic (e.g., congenital diaphragmatic hernia), urologic, and neurologic anomalies (e.g., myelomeningocele). The distribution of congenital anomalies included cardiac (50%, *n* = 8), surgical (25%, *n* = 4), neurosurgical (12.5%, *n* = 2), and other (12.5%, *n* = 2; both cases were twin–twin transfusion syndrome). Overall, 56% ( *n* = 9) of infants underwent postnatal surgical repair.

The cesarean section rate was 62.5% ( *n* = 10). Among these, half (50%, *n* = 5) of the infants delivered by cesarean section required postnatal surgery. Several infants had complex, multisystem diagnoses requiring early surgical intervention and prolonged hospitalization.

The study population reflected a racially and ethnically diverse group, with 44% identifying as African American ( *n* = 7), 31% as Caucasian ( *n* = 5), and 25% as Hispanic ( *n* = 4). Most mothers were multiparous (69%, *n* = 11) and publicly insured (75%, *n* = 12).

**Fig. 2:**
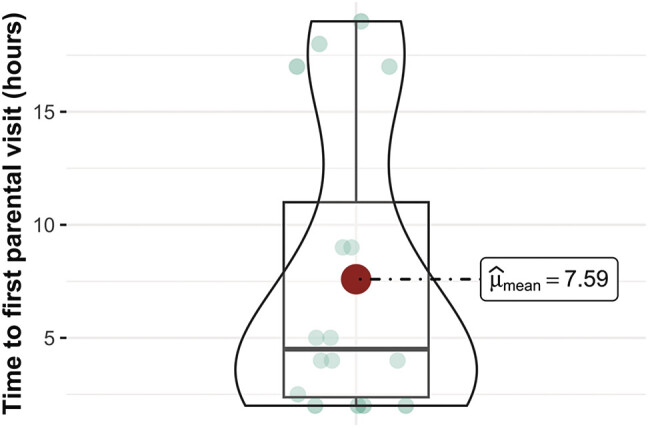
Distribution of time to first parental visit. The violin plot embedded boxplot displays the full distribution, including density, median, and interquartile range. The mean to first parental visit is indicated by the filled circle.

The mean time to first parental visitation was 7.6 h (±6.4), with a median of 4.5 h (interquartile range [IQR]: 2.4–11; Fig. 2 ). The mean time to first STS contact was 8.3 days (±7.4), with a median of 7 days (IQR: 2–15; Fig. 3 ). As shown in Table 1 , exploratory differences in time to first parental visit were observed across racial groups, with African American parents demonstrating earlier visitation at 3.5 ± 2.7 h, followed by Caucasian parents at 9.4 ± 6.9 h, and Hispanic parents at 12.5 ± 7.2 h (ANOVA, *p* = 0.05; Kruskal–Wallis sensitivity analysis *p* = 0.032; *n* = 16). The 95% CI for the differences in mean time were −15.68 to −2.32 h between African American and Hispanic parents, −12.23 to 0.43 h between African American and Caucasian parents, and −14.32 to 8.12 h between Caucasian and Hispanic parents. Descriptively, English-speaking parents engaged in STS earlier, at 6.2 days (± 6.5), compared with 17.5 days (± 0.7) for Spanish-speaking parents (not tested; *n* < 4 in one group). Privately insured families initiated STS at 5.6 ± 6.7 days, while publicly insured families began at 15.3 ± 3.8 days (not tested; *n* < 4 in one group; Table 2 ). Four English-speaking and one Spanish-speaking parent lacked STS documentation in the EMR.

**Fig. 3:**
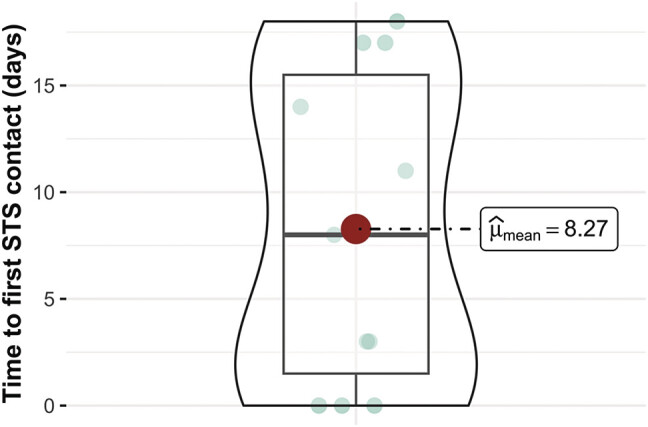
Distribution of time to first skin-to-skin contact. The violin plot embedded boxplot displays the full distribution, including density, median, and interquartile range. The mean to first skin-to-skin is indicated by the filled circle.

**Table 1:** Bivariate associations between time to first parental visit and selected maternal and infant characteristics ( *n* = 16).

Characteristics	*N*	Time to first parental visit in hours, mean (SD)	*p* -Value
Race			
African American	7	3.5 (2.7)	0.05 ^a^
Caucasian	5	9.4 (6.9)	
Hispanic	4	12.5 (7.2)	
EBM PD 14			
Yes	11	6.7 (2.9)	^b^
No	2	18.5 (0.7)	

Abbreviations: ANOVA, analysis of variance; CI, confidence interval; EBM PD 14, expressed breast milk on postnatal day 14; SD, standard deviation.

^a^ One-way ANOVA; 95% CIs for the difference in mean time: −15.68 to −2.32 h (African American vs. Hispanic), −12.23 to 0.43 h (African American vs. Caucasian), and −14.32 to 8.12 h (Caucasian vs. Hispanic); Kruskal–Wallis sensitivity analysis *p* = 0.032.

^b^ Not tested: sparse data ( *n* < 4) in at least one group; descriptive statistics only.

**Table 2:** Descriptive time to first skin-to-skin contact by selected sociodemographic characteristics.

Characteristic	*N*	Time to first skin-to- skin in days, mean (SD)
Language		
English	9	6.2 (6.5)
Spanish	2	17.5 (0.7)
Insurance		
Private	8	5.6 (6.7)
Public	3	15.3 (3.8)

Abbreviations: STS, skin-to-skin contact; SD, standard deviation.

Note: Statistical tests were not performed due to sparse data ( *n* < 4) in at least one group.

The distribution of time to first parental visit and time to first STS are shown in Figs. 2 and 3 , respectively. Violin plots with embedded boxplots illustrate the full distribution of each outcome, including density and IQR, with the mean highlighted to reflect the primary analytic approach. Both outcomes demonstrated right-skewed distributions, consistent with variability expected in a small, heterogeneous cohort.

EBM provision at postnatal day 14 was observed in 62.5% of cases ( *n* = 11). Infants receiving EBM had shorter times to first parental visit (6.7 ± 2.9 h) than those who did not receive EBM (18.5 ± 0.7 h; not tested; *n* < 4 in one group; Table 1 ). Earlier parental visitation and earlier initiation of STS contact were observed within the same cohort; however, the relationship between these outcomes was not formally evaluated.

MMH variables in this study were operationalized using clinically documented indicators, including prescription of antidepressant or anxiolytic medications and referral to perinatal psychiatry services. MMH challenges were prevalent. Of the 16 mothers, 6 (37.5%) were prescribed antidepressant or anxiolytic medication during or after delivery, and 5 (31.2%) were referred to perinatal psychiatry ( Table 3 ). Mothers with documented psychotropic medication prescriptions had longer mean times to first visitation (11.7 ± 6.9 h) compared with 5.2 ± 5.0 h in mothers without documented psychotropic medication prescriptions ( *p* = 0.046; 95% CI in mean difference: 0.14– 12.89 h; Table 4 ). This difference was borderline on Wilcoxon’s testing ( *p* = 0.055), and we therefore interpret it as suggestive.

**Table 3:** Maternal mental health characteristics in the postpartum period.

Characteristic	*N* = 16
Mother prescribed depression or anxiety medication	
Yes	6 (37.5%)
No	10 (62.5%)
Mother referred to perinatal psychiatrist	
Yes	5 (31.2%)
No	11 (68.6%)

**Table 4:** Maternal mental health characteristics and time to first parental visit.

Characteristic	*N*	Time to first parental visit in hours, mean (SD)	*p* -Value
Mother prescribed depression or anxiety medication			
Yes	6	11.7 (6.9)	0.046 ^a^
No	10	5.2 (5.0)	

Abbreviations: CI, confidence interval; SD, standard deviation.

^a^*t* -test; 95% CI in mean difference: 0.14 to 12.89 h (yes vs. no); Wilcoxon’s rank-sum sensitivity analysis *p* = 0.055.

## Discussion

This exploratory pilot study identified preliminary patterns suggesting that parental engagement among FC families may be influenced by psychosocial and structural factors in addition to infant medical acuity. 5 7 11 Medical characteristics, including respiratory support, hemodynamic instability, central lines, IVH, and postnatal surgical intervention, were not observed to be associated with differences in early visitation, STS timing, or EBM provision, suggesting that clinical severity alone may not suffice to explain patterns of parental participation.

Exploratory differences in visitation and STS timing were observed across language, insurance status, and MMH-related factors. Spanish-speaking and publicly insured families demonstrated longer times to STS contact, while mothers prescribed psychotropic medication showed longer delays to their first visit. Early visitation was also associated with EBM provision, supporting prior work linking parental presence with caregiving engagement and bonding behaviors. Together, these findings may be associated with variation in early parental participation in FC populations. 11 15

This study builds on existing NICU literature, which has primarily focused on preterm infants or infants affected by hypoxic–ischemic encephalopathy, by examining engagement patterns among families followed through an FC. 11 20 Families in this setting often begin their NICU experience during prenatal counseling and delivery planning, which introduces stressors distinct from those encountered in more traditional NICU populations. 5 22 Despite increasing integration of FCs into high-risk perinatal care, the postnatal experiences of FC families remain understudied. Prenatal diagnosis may heighten anticipatory grief, uncertainty, and emotional distress, potentially compounding the relational and logistical challenges of NICU hospitalization. To our knowledge, no published studies have systematically examined parental experience across the continuum from prenatal counseling in an FC to postnatal NICU care, and few have framed NICU-based interventions within this broader trajectory. The exploratory findings from this pilot study support the need for larger prospective studies to better characterize parental engagement across the prenatal-to-postnatal continuum and identify opportunities for targeted intervention in this understudied population. 5 14 22

These prenatal-to-postnatal stressors intersect with what Sanders and Hall describe as the biologic disruption inherent to the NICU. 7 Environmental stimuli, medical routines, and a limited structural emphasis on the parent–infant relationship may interfere with early attachment processes and relational health. 7 10 For families followed through an FC, these challenges may be further compounded by prolonged anticipatory stress that begins with prenatal diagnosis and extends through neonatal intervention. 5 6 The cumulative emotional burden of navigating diagnostic uncertainty, potential loss, and NICU hospitalization may contribute to delayed parental engagement or caregiver withdrawal, particularly among families with fewer resources or higher psychosocial risk. 8 14 Our findings are consistent with these relational dynamics and point to opportunities for earlier, targeted support to mitigate disparities in early engagement.

Delays in visitation and STS contact among Spanish-speaking parents are consistent with prior research demonstrating that language differences reduce communication quality, shared decision-making, and trust between clinicians and families. Even when interpretation services are available, logistical barriers and limited continuity may interfere with relationship building. Delays among publicly insured families likely reflect broader socioeconomic constraints such as lack of paid leave, limited access to transportation, or absence of childcare, factors known to influence NICU visitation patterns and EBM provision. These findings support prior studies linking systemic vulnerability to reduced engagement in NICU care and reinforce the need for family-centered procedures that accommodate varying levels of need and access. 4 23 Given the small sample size, variability and right-skew in time-to-event outcomes should be interpreted in context, but the observed patterns are consistent with prior work demonstrating structural barriers to parental engagement.

The association between earlier visitation and EBM provision supports existing literature suggesting that breast milk may serve as a marker of both parental presence and caregiving action. 19 23 Although breastfeeding support is integrated into NICU care at CMHH, our findings indicate that enhanced early lactation support, particularly for families facing systemic barriers, may strengthen parental identity, foster connection, and promote greater bedside consistency. 23 Notably, approximately one-third of infants in this cohort did not receive STS within the first month, highlighting a critical window to improve support and access to early bonding practices. 24

MMH appeared to be associated with early engagement patterns in this exploratory cohort. Mothers prescribed antidepressants or anxiolytics experienced longer delays in their initial NICU visitation, consistent with prior studies linking maternal psychological distress to lower engagement in care and poorer developmental outcomes in infants. 11 16 20 Maternal anxiety and depressive symptoms during the NICU hospitalization are associated with decreased visitation, reduced STS contact, and lower likelihood of sustained breast milk supply. 16 19 Mental health conditions may also impair a caregiver’s ability to recognize and respond to infant cues, reducing opportunities for co-regulation, connection, and relational scaffolding. 11 16 Maternal stress has additionally been linked to infant physiologic disruption and psychological changes, including altered autonomic regulation and elevated cortisol levels, which may influence long-term neurodevelopmental trajectories. 8 These findings support the potential value of early, integrated mental health screening and support pathways within neonatal care models, beginning in the FC and continuing through discharge. 18

Exploratory differences in visitation and STS practices were observed across language, insurance status, and MMH-related factors. These variables represent modifiable targets for future intervention. Expanding bedside access to language interpretation, childcare services, transportation support, and universal MMH screening may reduce these barriers and promote equitable access to early parent–infant bonding practices. Policies that integrate lactation and mental health services into the NICU standard of care may further strengthen and sustain parental engagement across diverse populations. 18 19

Strengths of this study include its mixed prospective–retrospective design, prospective real-time bedside tracking of parental presence, and focus on a historically understudied FC NICU population. By integrating dimensions of parental presence, lactation, and MMH, this study offers a new perspective on the complex dynamics shaping early dyadic engagement in FC families.

Several limitations also warrant consideration. The small sample size and single-site setting limit the generalizability of findings. The use of bedside calendars introduces potential for reporting bias, and the absence of longitudinal follow-up prevents assessment of long-term developmental and relational outcomes. Incomplete bedside documentation contributed substantially to study attrition and may have introduced selection bias. As a result, findings should be interpreted cautiously. Psychotropic medication prescriptions and psychiatry referrals were used as surrogate clinical indicators of MMH burden and may not fully capture the complexity, severity, or timing of maternal psychological distress. In addition, the lack of parental perspectives limits our ability to determine whether families perceive or prioritize the same barriers identified in clinician-collected data. Maternal and infant clinical factors, such as cesarean delivery and postnatal surgery, were reported but not fully examined as predictors of early engagement. The feasibility and timing of STS care may also have been influenced by infant medical stability, including postoperative status, respiratory support requirements, or other clinical limitations affecting early handling. Other maternal complications that may influence early engagement were not systematically captured. Missing subgroup data for language and insurance may have also constrained analysis. Although cumulative bedside presence was recorded when available, incomplete longitudinal bedside documentation limited reliable assessment of consistency of visitation and STS practices across full hospitalization. Therefore, analyses focused primarily on early engagement measures, including time to first visitation and first STS contact.

## Conclusion

This study contributes preliminary insight into the multifactorial barriers shaping parental engagement among families followed through an FC. Differences in visitation patterns, timing of STS contact, and MMH-related factors were observed and may reflect broader structural inequities within neonatal care systems. Exploratory findings suggest that modifiable psychosocial and systems-level factors may contribute to variation in early parental presence alongside infant medical characteristics. These results highlight the importance of neonatal care models that integrate early MMH screening, accessible lactation support, and flexible, family-centered practices to promote consistent and inclusive bedside engagement.

A follow-up study using a larger and more diverse cohort to validate these findings is warranted. As an exploratory pilot study, these findings support the feasibility of larger prospective studies designed to further evaluate barriers to parental engagement and test targeted interventions in FC populations. Future work should incorporate caregiver surveys or qualitative methods to assess parental perceived barriers, priorities, and forms of support. Understanding how institutional constraints are experienced within the family unit may inform the development of tailored interventions that are both acceptable and effective. These findings may help inform future prospective interventional studies, including factorial designs that examine the individual and combined effects of interventions such as early lactation support, integrated perinatal mental health services, and real-time language interpretation. Stratifying these trials by clinical or social risk profiles may enable targeted interventions to address inequities across NICU populations and build a more responsive, family-centered model of neonatal care in the FC population.
